# Ultrahigh-Resolution Photon-Counting CT in Cadaveric Fracture Models: Spatial Frequency Is Not Everything

**DOI:** 10.3390/diagnostics13101677

**Published:** 2023-05-09

**Authors:** Theresa Sophie Patzer, Andreas Steven Kunz, Henner Huflage, Nora Conrads, Karsten Sebastian Luetkens, Pauline Pannenbecker, Mila Marie Paul, Süleyman Ergün, Thorsten Alexander Bley, Jan-Peter Grunz

**Affiliations:** 1 Department of Diagnostic and Interventional Radiology, University Hospital Würzburg, Oberdürrbacher Straße 6, 97080 Würzburg, Germany; kunz_a@ukw.de (A.S.K.); huflage_h@ukw.de (H.H.); conrads_n1@ukw.de (N.C.); luetkens_k@ukw.de (K.S.L.); pannenbeck_p@ukw.de (P.P.); bley_t@ukw.de (T.A.B.); grunz_j@ukw.de (J.-P.G.); 2 Department of Orthopedic Trauma, Hand, Plastic and Reconstructive Surgery, University Hospital Würzburg, Oberdürrbacherstraße 6, 97080 Würzburg, Germany; paul_m1@ukw.de; 3 Institute of Anatomy and Cell Biology, University of Würzburg, Koellikerstraße 6, 97070 Würzburg, Germany; sueleyman.erguen@uni-wuerzburg.de

**Keywords:** photon-counting, tomography, X-ray computed, fracture, cancellous bone, convolution kernel

## Abstract

In this study, the impact of reconstruction sharpness on the visualization of the appendicular skeleton in ultrahigh-resolution (UHR) photon-counting detector (PCD) CT was investigated. Sixteen cadaveric extremities (eight fractured) were examined with a standardized 120 kVp scan protocol (CTDI_vol_ 10 mGy). Images were reconstructed with the sharpest non-UHR kernel (Br76) and all available UHR kernels (Br80 to Br96). Seven radiologists evaluated image quality and fracture assessability. Interrater agreement was assessed with the intraclass correlation coefficient. For quantitative comparisons, signal-to-noise-ratios (SNRs) were calculated. Subjective image quality was best for Br84 (median 1, interquartile range 1–3; *p* ≤ 0.003). Regarding fracture assessability, no significant difference was ascertained between Br76, Br80 and Br84 (*p* > 0.999), with inferior ratings for all sharper kernels (*p* < 0.001). Interrater agreement for image quality (0.795, 0.732–0.848; *p* < 0.001) and fracture assessability (0.880; 0.842–0.911; *p* < 0.001) was good. SNR was highest for Br76 (3.4, 3.0–3.9) with no significant difference to Br80 and Br84 (*p* > 0.999). Br76 and Br80 produced higher SNRs than all kernels sharper than Br84 (*p* ≤ 0.026). In conclusion, PCD-CT reconstructions with a moderate UHR kernel offer superior image quality for visualizing the appendicular skeleton. Fracture assessability benefits from sharp non-UHR and moderate UHR kernels, while ultra-sharp reconstructions incur augmented image noise.

## 1. Introduction

The visualization of the appendicular skeleton represents a central imaging task in musculoskeletal radiology. To warrant high diagnostic accuracy in the context of trauma, a dedicated depiction of the bone microarchitecture is mandatory for the detection of subtle pathologies like fine trabecular fissures and for the preoperative assessment of fracture morphology. In particular, the extent of the fragment dislocation as well as the involvement of adjacent structures and joint surfaces are of decisive relevance for preoperative planning. Owing to its cost-effectiveness and relatively low radiation burden, digital radiography continues to be the first choice in the workup of suspected injuries of the appendicular skeleton. As the detection of discrete pathologies remains challenging in standard 2D examinations due to the lack of superposition-free visualization, supplemental cross-sectional imaging is inevitable in some circumstances. Nonetheless, the penalty of a substantially increased radiation dose should be taken into account with regards to CT imaging. According to the ALARA principle (as low as reasonably achievable), the benefits and associated drawbacks of an additional CT examination must be critically discussed, particularly in young, and thus vulnerable, patients or, e.g., in repeated examinations.

Up to now, due to ubiquitous accessibility and rapid scan times, CT scanners with energy-integrating detectors (EIDs) are mainly employed for cross-sectional trauma examinations. Due to the limited spatial resolution in regular scan mode and an increasing radiation burden in ultrahigh-resolution (UHR) scans owing to the need to employ an additional comb filter to narrow the detector aperture, radiation saving potential is limited for this scanner type [1,2]. With increased image contrast and higher geometric dose-efficiency, photon-counting detectors (PCDs) offer substantial advantages over EIDs, improving the radiological assessment of fine bone structures at least in theory [3]. While EIDs contain scintillator elements, PCDs employ semiconductors (e.g., cadmium telluride) instead. PCD cells are defined by an electric field between the cathode and a pixelated anode, thus overcoming constructional restrictions such as the necessity to employ optically opaque separation layers [4]. In EID-CT, the compulsory septa between the detector pixels are designed to reduce crosstalk. The inherent constructional restraints regarding separation layers result in a smaller active detector area with limited geometric efficiency and, ultimately, a higher radiation dose.

The only commercially available system to date allows for superior spatial resolution by a separate readout of smaller subpixels with a maximal in-plane resolution of 0.11 mm in UHR mode [5,6]. In PCD-CT, incoming X-ray photons are directly converted into electrical signals, rendering the additional transformation step into visible light unnecessary [7,8]. Signal intensities are proportional to every single photon’s energy, hence overcoming the down-weighting of low-energy photons traditionally hampering EID-CT [9,10]. Furthermore, in PCD-CT, only photons exceeding a predefined threshold are integrated, thus effectively excluding low-level electronic noise [11,12]. 

Different aspects of image reconstruction may have an impact on image quality and bone delineation in CT examinations. Apart from field of view, slice thickness and matrix size, convolution kernel sharpness is one of the most important reconstruction parameters [13,14]. Reformatting data with dedicated bone kernels sharpens the images due to enhancement of the edges of high-contrast structures. However, the higher spatial resolution associated with sharp bone kernels usually comes with the price of increased image noise. With the emergence of PCD-CT, the established tradeoff between sharpness and noise for fracture assessment needs to be re-evaluated: either PCDs are capable of handling very sharp bone kernels better than the prior generation of EID-CT systems due to their detector-based denoising in quantum iterative reconstructions, or the adequate delineation of fine bone structures is feasible with softer bone kernels due to the system’s superior inherent raw modulation transfer function (MTF). Depending on the clinical application, the choice of an appropriate kernel offers the potential for dose reduction. 

While an increasing number of studies confirm the advantages of PCD-CT over EID-CT for the depiction of bone microarchitecture, e.g., of the wrist [15,16], large joints [17], as well as the paranasal sinus and temporal bone [18,19], the effect of different MTF levels on the assessability of the appendicular skeleton’s microarchitecture in UHR mode has not been thoroughly evaluated thus far. 

With the aim to fill this research gap, the present study employed various cadaveric fracture models to investigate the impact of convolution kernel sharpness in potential trauma settings.

## 2. Material and Methods

### 2.1. Cadaveric Fracture Models

**Figure 1:**
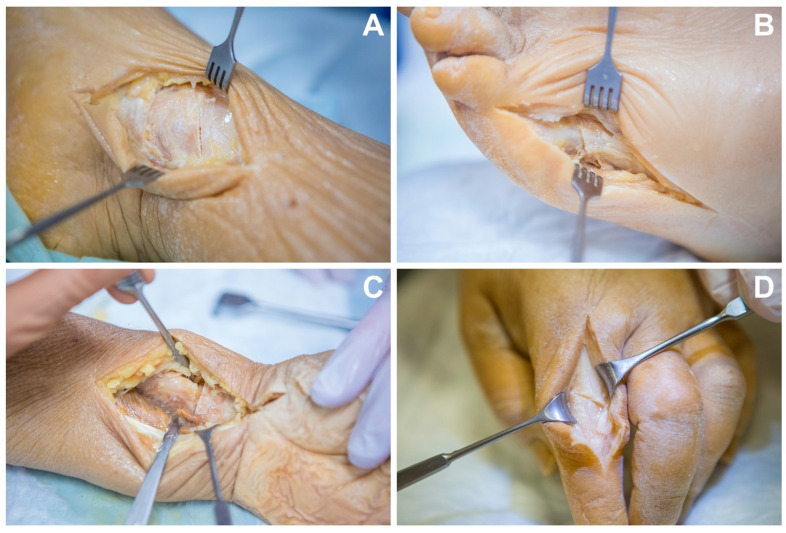
Cadaveric fracture models. Through a surgical access point, a board-certified trauma surgeon induced fractures by performing sequential osteotomies with an oscillating saw. Fractures were generated in different anatomic regions: (**A**) distal tibia, (**B**) metatarsal bone, (**C**) distal radial bone, (**D**) metacarpal bone.

Four formalin-fixed specimens were obtained from the local university’s anatomical institute. This experimental study was approved by the institutional review board and conducted in accordance with institutional laws and regulations. Individuals had donated their remains for study and research purposes during their lifetime, hence additional written informed consent was not required. In two cadaveric specimens, a board-certified trauma surgeon induced fractures of the distal radial bone, second metacarpal bone, distal tibia, distal fibula and fifth metatarsal bone. Through a surgical access point befitting the respective anatomical region, fractures were simulated by performing sequential osteotomies with an oscillating saw (Figure 1). Fracture regions were chosen for the reason that suspected injuries of these areas are among the most common imaging tasks and findings in emergency departments. Due to the in-part minute size of bones of the distal extremities, detailed visualization of bone microarchitecture is of great importance, especially for these body regions. Additionally, the dedicated depiction of fracture morphology is of high clinical relevance, in particular for preoperative planning in the joint areas (especially of the ankle and wrist). 

### 2.2. Imaging and Postprocessing

A total of eight fractured and eight non-fractured anatomical regions were included in the image analysis. Wrists, ankles, hands and feet were examined on a first-generation PCD-CT system (Naeotom Alpha; Siemens Healthcare GmbH, Forchheim, Germany). Scans of the lower extremity were performed in a supine position while the upper extremity was scanned in a “superman stance”, referring to the specimens being brought into a prone position with the respective arm above head level in the isocenter of the gantry. All examinations were conducted in standard polyenergetic scan mode (T3D) at 120 kVp, with a CTDI_vol_ of 10 mGy and UHR collimation of 120 × 0.2 mm. The field of view was chosen according to the clinical standard and an adapted matrix size was activated. Reformatting was performed in axial, coronal and sagittal orientations with an increment and slice thickness of 0.2 mm, i.e., the lowest slice thickness available in UHR mode. The image reconstruction included the sharpest non-UHR kernel (Br76) as well as all six available UHR-kernels (Br80, Br84, Br89, Br92, Br96, Br98). The spatial frequency of each kernel at different portions of the signal MTF is shown in Table 1. The reformation of the data was carried out with a fourth-generation quantum iterative reconstruction algorithm (QIR; Siemens Healthcare GmbH; strength level 3 of 4). The standard window width and center were predefined to 1500 and 450 Hounsfield units (HU), permitting an alteration of the window settings during the subjective image analysis.

### 2.3. Objective Image Quality

For quantitative analyses, a reader with three years of experience in musculoskeletal imaging placed circular regions of interest (ROI) within the cancellous bone of the distal radial and tibial bone, as well as in the subcutaneous fat tissue. ROI placement was conducted on Br76 images and copied to equivalent image positions in all other series. ROI size was predefined to 10 mm^2^. To calculate the signal-to-noise ratios (SNRs), the mean attenuation and standard deviation were recorded within each ROI, with the latter being considered representative of image noise.

### 2.4. Subjective Image Quality

The qualitative image assessment was independently performed by seven radiologists with two to nine years of skeletal imaging experience. For each region, the seven image reconstructions, i.e., one for each convolution kernel, were presented in a side-by-side fashion and randomized order. Image analysis was performed using dedicated PACS software (Merlin, Phönix-PACS, Freiburg im Breisgau, Germany) and certified diagnostic monitors (RadiForce RX660, EIZO, Hakusan, Ishikawa, Japan). Blinded to all protocol-related information, readers were asked to rank the reconstructions from 1 to 7, with “1” being considered to feature the best and “7” as the poorest image quality. Overall image quality analysis comprised the extent of noise, artifacts and depiction of soft tissue, as well as assessability of bone structures. Furthermore, the reconstructions were reviewed for assessability or the exclusion of fractures in a similar fashion.

### 2.5. Statistical Analysis

Dedicated software (SPSS Statistics 28, IBM, Armonk, NY, USA) was used for the statistical analyses. Employing a rank scale to investigate differences in image quality and fracture assessability, Friedman’s rank-based analysis of variance was used for comparisons between the various kernels. Pairwise post-hoc tests were Bonferroni-corrected for multiple comparisons. Data analysis of the parametric variables comprised the Shapiro–Wilk test for assessment of normal distribution and the Friedman test was also used for non-normally distributed parametric variables. To determine the degree of interrater reliability, the intraclass correlation coefficient was calculated based on a two-way random effects model for absolute agreement. *p* values ≤  0.05 were considered statistically significant.

## 3. Results

### 3.1. Objective Image Quality

**Figure 2:**
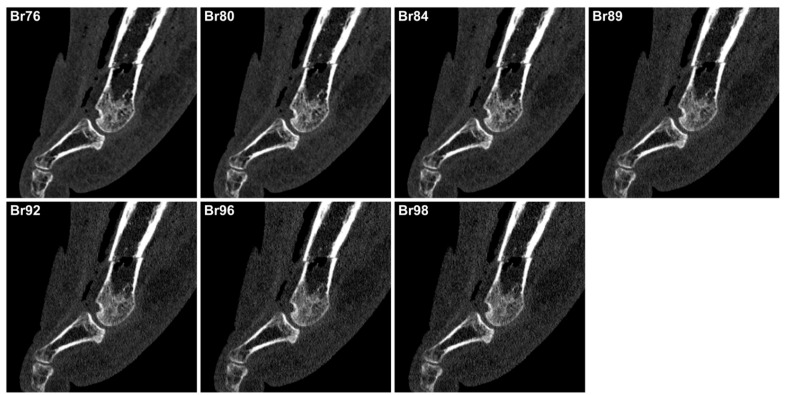
Image quality. Image quality associated with sharp non-UHR and all six available UHR kernels on representative sagittal slices depicting a midtarsal fracture. Importantly, sharper UHR kernels induced amplified image noise, which is particularly visible in soft tissue areas. Elevated noise levels were found to impair osseous assessability to a certain extent.

The signal and noise characteristics are summarized in Table 2. The highest SNR values were recorded for the non-UHR kernel Br76 (median 3.4, interquartile range 3.0–3.9) with no significant difference compared to the UHR kernels Br80 (3.4, 2.6–3.7) and Br84 (3.1, 2.2–3.4; *p* > 0.999). Br76 and Br80 kernels produced substantially higher SNRs than all UHR kernels sharper than Br84 (all *p* ≤ 0.026). The SNR difference between Br84 and the sharper Br89 (2.6, 2.0–3.1; *p* > 0.999) and Br92 kernels (2.4, 2.0–3.1; *p* = 0.077) was not significant. Figure 2 illustrates image signal and noise on representative sagittal slices reconstructed with different kernels.

### 3.2. Subjective Image Quality

Overall, the image quality was considered best for the Br84 reconstructions (median rank 1, interquartile range 1–3). The Br84 images were deemed superior compared to Br76 (3, 2–4, *p* = 0.003) and Br80 reformations (3, 2–4, *p* = 0.001), as well as compared to all the sharper UHR reconstructions (all *p* < 0.001). While the image quality of Br89 was comparable to Br76 (*p* = 0.866) and Br80 (*p* > 0.999), all UHR kernels with higher MTF than Br89 were deemed inferior to these two kernels (*p* < 0.001). Table 3 illustrates the results of the subjective image analysis.

**Figure 3:**
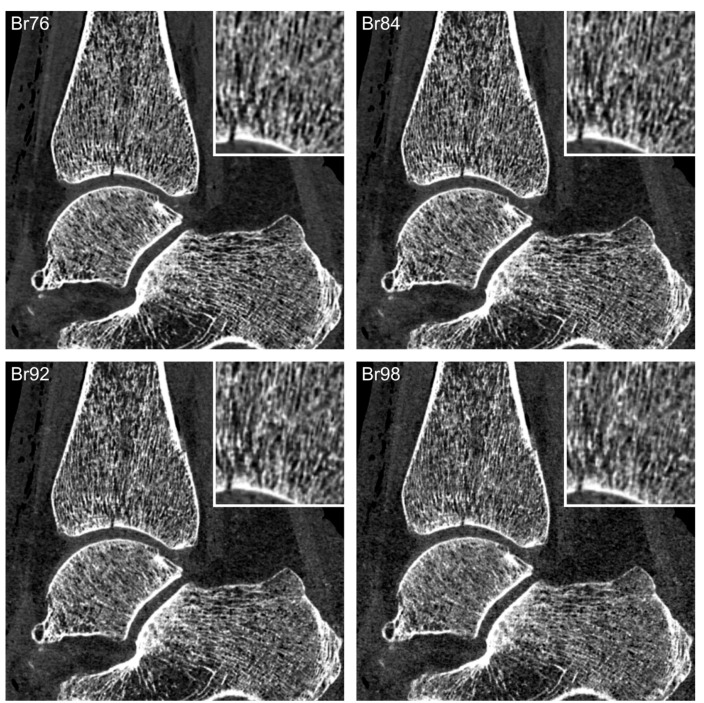
Articular fracture of the distal tibial bone. Fracture assessability is superior in sharp non-UHR (**Br76**) and moderate UHR kernels (**Br84**) compared with sharper UHR kernels (**Br92** and **Br98**).

Regarding fracture assessability, no significant difference was ascertained between sharp non-UHR reformatting (Br76) and the images reconstructed with moderate UHR kernels (Br80 and Br84; all *p* > 0.999). However, readers attributed superior fracture assessability to datasets reconstructed with each of these three kernels compared to all reconstructions performed with sharper convolution kernels (*p* < 0.001). It is of note that the two sharpest UHR kernels received the lowest ranks regarding both image quality and fracture assessability (Br96: 6, 6–6; Br98: 7, 6–7; both *p* ≥ 0.932). Figure 3 depicts the fracture assessability in images reconstructed with different kernels.

Interrater reliability for overall image quality and fracture assessability was good, indicated by intraclass correlation coefficient values of 0.795 (95% confidence interval 0.732–0.848; *p* < 0.001) and 0.880 (0.842–0.911; *p* < 0.001), respectively.

## 4. Discussion

With the aim to investigate the impact of convolution kernel selection on the fracture diagnosis of the appendicular skeleton, this study comprised qualitative and quantitative image quality analyses in cadaveric fracture models on a first-generation photon-counting CT. With superior ratings for sharp non-UHR and moderate UHR kernels, our multi-observer analysis revealed that kernel sharpness may not be the deciding factor for fracture assessability. Instead, the decreased image noise associated with a lower modulation transfer function proved beneficial for subjective image assessment. 

In the wake of PCD-CT introducing dose-neutral full field of view UHR imaging, the influence of proper kernel selection was recently investigated for different imaging tasks, e.g., in lung [20,21] and stent imaging [22] as well as in coronary CT angiography [23]. However, the effect of MTF variation on bone delineation—arguably the original domain of UHR imaging—has not been thoroughly investigated so far. To exploit the full potential of the PCD technology, a plethora of ultra-sharp convolution kernels are available for image reconstruction. However, despite complementing the detector’s maximum in-plane resolution of 125 μm [24], both image quality and fracture assessability were rated low for the two sharpest UHR kernels (Br96 and Br98) with the applied dose of 10 mGy. While somewhat surprising, we believe that this finding may be attributed to the increased image noise resulting from both kernels’ higher spatial frequencies combined with the fairly low radiation exposure in this study [25,26]. Accordingly, we report better SNRs in this study for the moderately sharp Br84. The results of our multi-observer analysis even indicated higher image quality for non-UHR reconstructions compared with UHR reconstructions performed with ultra-sharp convolution kernels. Achieving superior fracture assessability despite considerably lower kernel MTF could be associated with the high intrinsic spatial resolution of the PCD-CT scanner. Thus, we hypothesize that the benefit of an even higher spatial resolution with sharper image kernels may be offset when employing this detector build for fracture analysis. In essence, the downside of increased image noise with sharper kernels seems to outweigh the advantage of minimally improved spatial resolution at 10 mGy. Therefore, at this dose level, the tradeoff between noise and sharpness in PCD-CT appears to be optimal for a ρ_50_ value (spatial frequency where the MTF is 50% of the peak MTF) between 16.5 and 22.6 lp/cm, allowing for a detailed delineation of fine bone microarchitecture.

Leng at al. [5] demonstrated the benefit of small pixel size in UHR mode compared to imaging with standard pixel size, reporting an associated potential for image noise reduction. By maintaining the in-plane spatial resolution at a constant level, image noise was reduced up to 25% in UHR images. This fact is explained by the smaller pixel size in UHR mode with subsequent higher intrinsic MTF. Hence, a more potent smoothing filter can be employed in order to achieve similar final MTF values. This consequently leads to an amplified image noise reduction, potentially allowing for corresponding dose savings. In synopsis, Klein et al. postulated the advantages of using small pixel size and recommended avoiding the binning of detector cells to achieve the highest possible image quality and low radiation dose even in scenarios where high resolution is not necessarily required. The same group had previously described the noise reduction potential associated with acquiring small pixel images in UHR mode and reconstructing them with a lower MTF than the scanner resolution limit [27]. This phenomenon has recently gained recognition as the “small pixel effect” and becomes even more pronounced in comparison to conventional EID-based scanner systems [26]. For example, Rajendran et al. have reported a potential dose reduction of 82% for kernel-matched PCD-CT examinations compared to EID-CT [24]. This is in line with a study by Booij et al. investigating the visibility of bone structures in the wrist with a PCD-CT system [28]. While this study revealed that reconstructions with a very sharp kernel (Br92) offer superior image quality at half the dose of an EID-CT protocol (CTDI_vol_ 12.2 vs. 6.1 mGy), it did not investigate the effect of different convolution kernels. This aspect was recently analyzed by Kämmerling et al., however, who found superior image quality for bone structures of the wrist with sharper kernels, reduced slice thickness and larger image matrix size [29]. The authors postulated optimal trabecular assessment for reconstructions with the sharpest investigated UHR kernel (Br89). In contrast, we demonstrate the best fracture assessability of the appendicular skeleton with the smoother Br84 (CTDI_vol_ 10 mGy). It is noteworthy that the present study included images reconstructed with all available UHR kernels, whereas the maximum ρ_50_ value investigated in the only previous kernel comparison was 27.0 lp/cm. Furthermore, while Kämmerling et al. [29] focused on healthy cadaveric wrists, the current investigation additionally included surgical fracture models of the hand, ankle and foot aiming to evaluate the impact of kernel selection on different osseus regions to ensure the transferability of the results to the entire peripheral appendicular skeleton. Fracture models were intended to simulate a potential post-traumatic scenario, albeit with induced injuries of an artificial nature due to the study’s design. It should be taken into account that SNR is dose-dependent and therefore the selection of the most appropriate convolution kernel depends on the applied radiation dose. Hence, applying considerably higher doses may allow for superior results with sharper kernels.

Due to the inherent contrast in skeletal CT examinations, high frequency kernels are usually used for the evaluation of bone structures, tolerating increased image noise [4]. Divergent to this approach, Willaume et al. demonstrated superior detection of sacral stress fractures with smooth kernels in patients with demineralized bone material [30]. As formalin fixation invokes demineralization of the bone, this effect may have had a notable influence on our results as well. Considering the aging population of Western countries, the aspect of decreased bone quality in osteoporotic patients needs to be recognized when choosing the optimal acquisition and reconstruction parameters for fracture analysis.

### Limitations

Several limitations have to be discussed regarding this study. First, the appendicular skeletons of four formalin-fixated cadaveric specimens were examined. Despite including eight extremities without fractures and eight fractured extremities, the clinical transferability of the results may be somewhat limited since the fractures were surgically induced. Second, while this investigation focused on the visualization of bone structures, the evaluation of the adjacent soft tissue was not within the scope of this study and would have required an evaluation of dedicated soft tissue kernels. 

Third, only cadaveric specimens without metal implants were included in this study, thus the effect of different convolution kernels on artifact reduction could not be evaluated. As dedicated visualization of the bone, as well as the implant itself, is essential in postoperative assessment after osteosynthetic treatment of fractures to account for postoperative complications, further investigations are mandated to evaluate the effect of kernel selection on image quality in postoperative settings.

Fourth, the body donors’ age, bone density, pre-existing degenerative bone changes and duration of formalin fixation may have influenced image quality results [31,32]. Fourth, since SNR differs with radiation dose level, ideal kernel choice may be different for other clinical applications. Consequently, further studies are warranted to evaluate the impact of reconstructions with different kernels at various radiation dose levels as well as in clinical patient samples.

## 5. Conclusions

Photon-counting CT reconstructions with a moderate UHR kernel offer superior image quality for visualizing the appendicular skeleton. Fracture assessability benefits from sharp non-UHR and moderate UHR kernels, while ultra-sharp reconstructions incur augmented image noise with subsequently decreased discrimination of bone microarchitecture.

## Figures and Tables

**Table 1:** Kernel properties.

Kernel	Frequency at the 50% Value of the MTF (ρ_50_) [lp/cm]	Frequency at the 10% Value of the MTF (ρ_10_) [lp/cm]	Frequency of the Maximum of the MTF (ρ_max_) [lp/cm]
**Br76**	16.5	21.0	7.8
**Br80**	19.3	24.9	8.9
**Br84**	22.6	27.9	10.5
**Br89**	27.0	30.0	14.0
**Br92**	30.4	33.5	15.1
**Br96**	34.9	37.8	18.0
**Br98**	39.0	42.9	20.4

**Note:** All information according to vendor information. **MTF**—modulation transfer function.

**Table 2:** Signal and noise characteristics.

	Br76	Br80	Br84	Br89	Br92	Br96	Br98
**Image noise [HU]**	30.3 (27.5–35.5)	30.3 (27.7–39.0)	32.5 (29.7–41.7)	36.5 (34.7–49.6)	39.4 (35.9–51.0)	41.6 (36.5–55.9)	42.1 (36.6–48.7)
**Signal-to-noise ratio**	3.4 (3.0–3.9)	3.4 (2.6–3.7)	3.1 (2.2–3.4)	2.6 (2.0–3.1)	2.4 (2.0–3.1)	2.3 (1.9–3.0)	2.3 (1.8–3.0)

**Note**: **IQR**—interquartile range.

**Table 3:** Subjective image quality. Pooled ratings of seven radiologists for overall image quality and fracture assessability. Results are presented as absolute frequencies with percentages in parentheses.

	Image Quality							Fracture Assessability						
	Br76	Br80	Br84	Br89	Br92	Br96	Br98	Br76	Br80	Br84	Br89	Br92	Br96	Br98
**1**	23 (20.5)	5 (4.5)	66 (58.9)	2 (1.8)	0 (0)	0 (0)	16 (14.3)	48 (42.9)	5 (4.5)	47 (42.0)	1 (0.9)	0 (0)	2 (1.8)	9 (8.0)
**2**	27 (24.1)	39 (34.8)	12 (10.7)	18 (16.1)	1 (0.9)	15 (13.4)	0 (0)	19 (17.0)	44 (39.3)	25 (22.3)	15 (13.4)	2 (1.8)	6 (5.4)	1 (0.9)
**3**	25 (22.3)	36 (32.1)	17 (15.2)	16 (14.3)	17 (15.2)	1 (0.9)	0 (0)	25 (22.3)	40 (35.7)	25 (22.3)	7 (6.3)	10 (8.9)	3 (2.7)	2 (1.8)
**4**	18 (16.1)	14 (12.5)	4 (3.6)	55 (49.1)	21 (18.8)	0 (0)	0 (0)	15 (13.4)	16 (14.3)	4 (3.6)	63 (56.3)	10 (8.9)	2 (1.8)	2 (1.8)
**5**	4 (3.6)	3 (2.7)	11 (9.8)	19 (17.0)	68 (60.1)	6 (5.4)	1 (0.9)	1 (0.9)	3 (2.7)	2 (1.8)	17 (15.2)	77 (6.9)	10 (8.9)	2 (1.8)
**6**	2 (1.8)	12 (10.7)	2 (1.8)	2 (1.8)	4 (3.6)	76 (6.8)	26 (23.2)	1 (0.9)	3 (2.7)	3 (2.7)	6 (5.4)	9 (8.0)	76 (67.9)	14 (12.5)
**7**	13 (11.6)	3 (2.7)	0 (0)	0 (0)	1 (0.9)	14 (12.5)	69 (61.6)	3 (2.7)	1 (0.9)	6 (5.4)	3 (2.7)	4 (3.6)	13 (11.6)	82 (73.2)
**Median** **(IQR)**	**3** **(2–4)**	**3** **(2–4)**	**1** **(1–3)**	**4** **(3–4)**	**5** **(4–5)**	**6** **(6–6)**	**7** **(6–7)**	**2** **(1–3)**	**3** **(2–3)**	**2** **(1–3)**	**4** **(4–4)**	**5** **(5–5)**	**6** **(6–6)**	**7** **(6–7)**

**Note**: **IQR**—interquartile range.

## Data Availability

Data is made available upon reasonable request.

## Abbreviations

CTDI_vol_	volume computed tomography dose index
EID	energy-integrating detector
MTF	modulation transfer function
PCD	photon-counting detector
ROI	region of interest
SNR	signal-to-noise ratio
UHR	ultrahigh-resolution
